# Shear Bond Strengths of Different Adhesive Systems to Biodentine

**DOI:** 10.1155/2013/626103

**Published:** 2013-10-10

**Authors:** Mesut Enes Odabaş, Mehmet Bani, Resmiye Ebru Tirali

**Affiliations:** ^1^Department of Pediatric Dentistry, Faculty of Dentistry, University of Gazi, 8 Cadde, 82 Sokak, 06510 Ankara, Turkey; ^2^Department of Pediatric Dentistry, Faculty of Dentistry, University of Başkent, 06490 Ankara, Turkey

## Abstract

The aim of this study was to measure the shear bond strength of different adhesive systems to Biodentine with different time intervals. Eighty specimens of Biodentine were prepared and divided into 8 groups. After 12 minutes, 40 samples were randomly selected and divided into 4 groups of 10 each: group 1: (etch-and-rinse adhesive system) Prime & Bond NT; group 2: (2-step self-etch adhesive system) Clearfil SE Bond; group 3: (1-step self-etch adhesive systems) Clearfil S^3^ Bond; group 4: control (no adhesive). After the application of adhesive systems, composite resin was applied over Biodentine. This procedure was repeated 24 hours after mixing additional 40 samples, respectively. Shear bond strengths were measured using a universal testing machine, and the data were subjected to 1-way analysis of variance and Scheffé post hoc test. No significant differences were found between all of the adhesive groups at the same time intervals (12 minutes and 24 hours) (*P* > .05). Among the two time intervals, the lowest value was obtained for group 1 (etch-and-rinse adhesive) at a 12-minute period, and the highest was obtained for group 2 (two-step self-etch adhesive) at a 24-hour period. The placement of composite resin used with self-etch adhesive systems over Biodentine showed better shear bond strength.

## 1. Introduction

Mineral Trioxide Aggregate (MTA) has rapidly gained acceptance in dentistry since its introduction in 1993 by Torabinejad [1]. However, MTA displays important limitations such as extending time, difficult handling properties, and discoloration potential of dental tissue [2, 3]. Recently, Biodentine (Septodont, Saint-Maur-des-Fossés, France) is a new tricalcium silicate-based restorative material has been introduced. The main component of Biodentine powder is tricalcium silicate, with the addition of calcium carbonate and zirconium oxide. The liquid is a solution of calcium chloride with a water-reducing agent. The addition of calcium chloride results in shorter setting time, as it also accelerates the rate of early strength development. Thus, the main advantages of Biodentine over MTA are its greater viscosity and its shorter setting time (12 min approximately).

Recently, promising biological properties have been reported for Biodentine on human pulp fibroblast cultures and on the dental pulp in an entire human tooth culture model [4, 5]. It has been demonstrated that Biodentine induced an effective dentinal repair when applied directly to mechanically exposed rat pulps [6]. In addition, Nowicka et al. [7] evaluated the clinical, radiographical, and histologic responses of the pulp-dentin complex after direct capping with Biodentine and MTA in human teeth. They found similar efficacy in the clinical setting and suggested that Biodentine may be considered an alternative to MTA in pulp-capping treatment during vital pulp therapy.

Biodentine is recommended for use as a dentine substitute under resin composite restorations and an endodontic repair material because of its biocompatibility, bioactivity, and biomineralization properties [4, 5]. However, the potential of restorative materials to attach Biodentine is not well known. The purpose of this study was to evaluate the shear bond strength of 3 different adhesive systems (etch-and-rinse adhesive, two-step self-etch adhesive, and one-step self-etch adhesive systems) to Biodentine.

## 2. Materials and Methods

Three commercial adhesive systems, (etch-and-rinse adhesive system) Prime & Bond NT, (two-step self-etch adhesive system) Clearfil SE Bond, and (one-step self-etch adhesive systems) Clearfil S^3^ Bond, were tested in this study and applied as recommended by the manufacturers. The materials used are listed in Table 1.

### 2.1. Specimen Fabrication

A total of 80 acrylic blocks containing a central hole with a 4 mm diameter and a 2 mm height were prepared. Biodentine was mixed according to the manufacturer's instructions. The acrylic blocks were fully filled with Biodentine. Then, the specimens were stored at 37°C with 100% humidity for 12 minutes and 24 hours to encourage setting.

 After 12 minutes, 40 samples were randomly selected and divided into 4 groups of 10 each: group 1: (etch-and-rinse adhesive system) Prime & Bond NT; group 2: (two-step self-etch adhesive system) Clearfil SE Bond; group 3: (one-step self-etch adhesive systems) Clearfil S^3^ Bond; group 4: control (no adhesive). In groups 1, 2, and 3, the corresponding adhesive system was applied over Biodentine according to the manufacturer's instructions (Table 1), whereas in group 4, no adhesive system was applied. A composite material (Clearfil Mejesty, Kuraray Noritake Dental Inc, Okayama, Japan) was applied into a cylindrical shaped plastic matrix with an internal diameter of 2 mm and a height of 2 mm. Light curing was administered with a light-emitting diode light-curing unit (Elipar FreeLight 2: 3M ESPE, St Paul, MN, USA) with an intensity of 1,200 mV/cm^2^ for 20 seconds. This procedure was repeated at 24 hours after mixing additional 40 samples, respectively.

### 2.2. Shear Bond Strength Test

The polymerized specimens were stored in 100% relative humidity at 37°C for 24 hours. For shear bond strength testing, the specimens were secured in a holder placed on the platen of the testing machine and then sheared with a knife-edge blade on a universal testing machine (Lloyd LRX: Lloyd Instruments, Fareham, Hants, UK) at a crosshead speed of 1.0 mm/min. Shear bond strength in MPa was calculated by dividing the peak load at failure with the specimen surface area.

*Fracture Analysis.* Fractured test specimens were examined under a stereomicroscope at a magnification of ×25 (Stemi 2000C: Carl Zeiss, Gottingen, Germany). Specimen fractures were classified as follows: cohesive failure exclusively within Biodentine, cohesive failure exclusively within restorative material, adhesive failure that occurred at the Biodentine-restorative material interface, or mixed failure when 2 modes of failure happened simultaneously. Fracture analysis was performed by a single observer who was completely uninformed about the experimental groups.

### 2.3. Statistical Analysis

One-way analysis of variance was used to detect differences in bond strength among the experimental groups. Post hoc comparisons were performed using the Scheffé test.

## 3. Results

The mean values and standard deviations of shear bond strengths are given in Table 2. 

When shear bond strengths of adhesive systems were compared, no significant differences were found between group 1 (etch-and-rinse adhesive), group 2 (two-step self-etch adhesive), and group 3 (one-step self-etch adhesive) at the same time intervals (*P* > .05). 

Among 2 time intervals, the bond strength of group 2 (two-step self-etch adhesive) at a 24-hour period presented significantly higher bond strength values (19,559 MPa) than group 1 (etch-and-rinse adhesive) and group 4 (control) at 12-minute period (*P* < .05). 

Table 3 shows the fracture modes of the experimental groups. Most of the observed modes of failure were cohesive in Biodentine and adhesive failure. No specimens failed cohesively within composite resin. 

## 4. Discussion

Because Biodentine is recommended for use as a dentine substitute under restorations, the bond strength between restorative materials and Biodentine is important for the quality of filling. In this study, the bond strength of a resin composite when bonded to Biodentine with 3 different adhesive systems (i.e., etch-and-rinse adhesive, two-step self-etch adhesive, and one-step self-etch adhesive systems) was evaluated at 2 time intervals (12 min and 24 h). We found that the mean bond strength values ranged from 9,127 to 19,559 MPa. The lowest value was obtained for group 1 (etch-and-rinse adhesive) at a 12-minute period, and the highest was obtained for group 2 (two-step self-etch adhesive) at a 24-hour period. Failure analysis showed adhesive, cohesive, and/or mixed fractures, depending on the adhesive tested. In this study, a general trend was observed; specimens that presented with lower bond strength failed more at composite resin and Biodentine interface (adhesive). On the other hand, specimens with higher bond strength failed more cohesively in Biodentine.

Tricalcium silicate is one of the main constituents of MTA. Tricalcium silicate is used as a bone cement [7] and also as an endodontic material [8]. This material is synthesized in the laboratory from high purity raw materials unlike Portland cements in MTA. It has been demonstrated that pure tricalcium silicate is a suitable replacement for the cementitious component in MTA due to their similar composition and bioactivity [9]. Tricalcium silicate cement has been found to have shorter setting time than MTA and good injectability and bioactivity [10]. One such formulation is Biodentine (Septodont) which was developed as dentin replacement material.

There are no studies evaluating the bond strength of restorative materials when bonded to Biodentine with adhesive systems for the purpose of outcome comparison. However, tricalcium silicate is the main component of MTA [11]; the outcomes of this study could be compared with earlier studies about MTA. 

Tunç et al. [12] evaluated the bond strength of a composite and a compomer to white MTA using etch-and-rinse adhesive (Single Bond) and one-step self-etch adhesive (Prompt L-Pop). Different from our findings, they found etch-and-rinse adhesive systems bonded to white MTA significantly more strongly than and one-step self-etch adhesive systems in both composite and compomer materials. Bayrak et al. [13] also found that etch-and-rinse adhesive systems exhibited higher shear bond strength than self-etch adhesive systems. However, Neelakantan et al. [14] found that one-step self-etch adhesive (Clearfil S^3^ Bond) demonstrated higher bond strength to white MTA than did the two-step self-etch adhesive (AdheSE) and the etch-and-rinse adhesive systems (Prime & Bond NT) immediately and 24 hours after fabrication. Researchers have not reached a consensus on appropriate adhesive systems over MTA yet.

In this study, the highest bond strength value was obtained with self-etch adhesive systems. There is controversy concerning the efficacy of self-etch systems. Some investigations show that they provide dentin bond strength comparable with that obtained with etch-and-rinse system [15–18], whereas others have observed significantly lower bond strengths [19–22]. Amongst the self-etch adhesives, the results of this study revealed that two-step self-etch adhesive system (Clearfil SE Bond) exhibited higher shear bond strength than one-step self-etch adhesive system (Clearfil S^3^ Bond). This result was in agreement with those of previous studies which found that the bond strengths of two-step self-etch adhesives were higher than those of one-step self-etch adhesives [23–26].

## 5. Conclusions

This in vitro study found no statistically significant differences between all the three adhesive systems at each of the 2 time intervals. However, Biodentine has shorter setting time than MTA (12 min); the highest bond strength value was obtained for two-step self-etch adhesive at a 24-hour period. On the other hand, because of the variations in the composition of different resin composites and adhesive systems, different results could be achieved. The adhesive system did not affect the bond strength of Biodentine. Further investigations are needed for better understanding of the adhesion mechanism of adhesive systems to Biodentine. 

## Figures and Tables

**Table 1:** Materials used in the study.

Material	Composition	Steps of application
Biodentine (Septodont, Saint-Maur-des-Fossés Cedex, France)	*Powder* Tricalcium silicate, dicalcium silicate, calcium carbonate and oxide, iron oxide, and zirconium oxide *Liquid* Calcium chloride and hydrosoluble polymer	Five doses liquid and powder supplied for 30 s with a mixed amalgamator

Composite (Clearfil Majesty, Kuraray Noritake Dental Inc., Okayama, Japan)	Silaned barium glass filler, prepolymerized organic filler, bisphenol A-glycidyl methacrylate (bis-GMA), hydrophobic aromatic dimethacrylate, and dicamphorquinone	Light-cure for 20 s

Prime & Bond NT (Caulk/Dentsply International Inc., Milford, DE, USA)	Di- and trimethacrylate resin, PENTA, functionalized amorphous silica, photoinitiators, stabilizers, cetylamine, hydrofluoride, and acetone	(1) Apply 35% phosphoric. acid etchant for 15 s. (2) Rinse and blot-dry. (3) Apply bond. (4) Allow gentle air stream. (5) Light-cure for 10 s.

Clearfil SE Bond (Kuraray Noritake Dental Inc, Okayama, Japan)	*Primer* 10-Methacryloyloxydecyl dihydrogen phosphate (MDP), HEMA, hydrophilic aliphatic dimethacrylate, dicamphoroquinone, N-diethyl-p-toluidine, and water *Bond* 10-Methacryloyloxydecyldihy drogenphosphate (MDP), bisphenol A-glycidyl methacrylate (bis-GMA), HEMA, hydrophobic aliphatic dimethacrylate, dicamphoroquinone, N-diethyl-p-toluidine, and colloidal silica	(1) Apply primer for 20 s. (2) Dry with mild air for 5 s. (3) Apply bond for 10 s. (4) Apply air flow gently (5) Light-cure for 10 s.

Clearfil S^3^ Bond (Kuraray Noritake Dental Inc, Okayama, Japan)	10-Methacryloyloxydecyldihy drogenphosphate (MDP), bisphenol A-glycidyl methacrylate (bis-GMA), HEMA, hydrophobic dimethacrylates, dicamphoroquinone, ethanol, water, and silanated colloidal silica	(1) Apply bond for 10 s. (2) Dry with mild air for 5 s. (3) Light-cure for 10 s.

**Table 2:** Mean shear bond strength values of adhesives (MPa) to Biodentine.

Composite	*N *	Mean ± SD	
		12 min	24 h
Group 1: Prime & Bond NT	10	9.127 ± 3.161^a^	15.990 ± 3.409^a,c^
Group 2: Clearfil SE Bond	10	16.903 ± 8.112^a,c^	19.559 ± 7.582^c^
Group 3: Clearfil S^3^ Bond	10	11.057 ± 3.850^a,c^	15.193 ± 3.344^a,c^
Group 4: Control	10	1.600 ± 0.512^b^	1.737 ± 0.434^b ^

Different superscript letters indicate significant differences by one-way ANOVA and post hoc Scheffé test within the same time interval or at different time intervals (*P* > .05).

**Table 3:** Fracture modes of thespecimens after shear bond test bond.

	Total	Prime & Bond NT		Clearfil SE Bond		Clearfil S^3^ Bond		No adhesive	
		12 min	24 h	12 min	24 h	12 min	24 h	12 min	24 h
Adhesive	38	4	2	3	2	4	3	10	10
Mixed	9	1	2	2	2	1	1	—	—
Cohesive in Biodentine	33	5	6	5	6	5	6	—	—
Cohesive in composite resin	0	—	—	—	—	—	—	—	—
